# Intra-Articular Calcaneal Fractures: Comparison between Mini-Invasive Approach and Kirschner Wires vs. Extensive Approach and Dedicated Plate—A Retrospective Evaluation at Long-Term Follow-Up

**DOI:** 10.3390/jcm12010020

**Published:** 2022-12-20

**Authors:** Silvio Caravelli, Giammarco Gardini, Camilla Pungetti, Paolo Gentile, Carlo Perisano, Tommaso Greco, Vito Gaetano Rinaldi, Giulio Maria Marcheggiani Muccioli, Domenico Tigani, Massimiliano Mosca

**Affiliations:** 1 II Clinic of Orthopaedic and Traumatology, IRCCS Istituto Ortopedico Rizzoli, 40136 Bologna, Italy; 2 U.O. Ortopedia e Traumatologia, Ospedale Maggiore “Pizzardi”, 40133 Bologna, Italy; 3 Orthopaedics and Trauma Surgery Unit, Department of Ageing, Neurosciences, Head-Neck and Orthopaedics Sciences, Fondazione Policlinico Universitario Agostino Gemelli IRCCS, 00168 Roma, Italy

**Keywords:** calcaneal fracture, K-wires, dedicated plate, complications, outcomes

## Abstract

Introduction: Calcaneal fractures (CF) are the most common tarsal fractures, representing up to 75% of foot fractures and 2% of all fractures. The aim of this retrospective study is to analyze fixation with Kirschner wires through a mini-invasive approach and dedicated plate and screws through an extended approach at long-term follow-up. Materials and Methods: Patients were radiographically and clinically evaluated at final follow-up, by using the validated American Orthopedic Foot and Ankle Society (AOFAS) Ankle-Hindfoot score for the clinical–functional assessment, the Short-Form 12 (SF-12) for the physical and psychological domain assessment, and the Visual Analog Scale (VAS) for pain. Results: In total, 38 patients (42 CF) met the inclusion criteria and were retrospectively evaluated and divided into two groups (Kirschner group and plate group) consisting of 19 patients each. The overall mean follow-up was 59.4 ± 11.8 months. The average values of the post-operative clinical outcomes of the two groups KG and PG were, respectively, 70.7 ± 11.9 and 70.1 ± 10.9 (AOFAS), 45.7 ± 6.8 and 46.5 ± 10.8 (SF-12 PCS), 54.7 ± 9.9 and 50.9 ± 11.8 (SF-12 MCS) at the final follow-up. Conclusions: The present study showed that in the cases analyzed, the two surgical approaches used for the treatment of CF achieved comparable clinical outcomes. The only substantial difference found between the two groups of patients was the re-intervention rate that afflicted them.

## 1. Introduction

Calcaneal fractures (CF) are the most common tarsal fractures, representing up to 75% of foot fractures and 2% of all fractures [1]. The etiology is to be found in high-energy axial traumas, generally falls from great heights. This type of fracture often has negative socio-economic consequences for the patient, being frequently associated with long-term morbidity and disability in complex fracture patterns.

The difficulties in the management of CF are reflected in various treatment modifications throughout the years. During the 18th and 19th centuries, prevention of life-threatening infections was the primary goal with this type of fracture. In 1920, some French surgeons developed the open reduction and internal fixation of displaced intra-articular calcaneus fractures with staples and screws and filled the bone defects with autologous bone grafts. In 1934, H. Westhues used a percutaneous pin in the tuberosity to reduce the main fragments and then he immobilized the foot in a plaster cast [2]. In 1938, Goff described, in a review, more than 41 different operative treatment methods for calcaneus fractures [3].

The gold standard in the treatment of intra-articular CF is now widely considered as open reduction and internal fixation with dedicated plates and screws. Other, different surgical techniques have, however, shown encouraging results and low rates of complications, including temporary fixation with K wires and minimally invasive access [4].

Over the past decade, research has focused mainly on comparative studies analyzing the dichotomy between the conservative and surgical management of these fractures, or the type of surgical access for the placement of dedicated plates or screws that would give better results [3,5,6,7,8].

The aim of this retrospective study is to analyze two different surgical techniques (fixation with Kirschner wires through mini-invasive sinus tarsi approach versus dedicated plate and screws through extended approach) for the treatment of intra-articular CF in terms of clinical and radiological outcomes.

## 2. Materials and Methods

This is an evidence level III, multicentric, retrospective, comparative study with a 1:1 allocation ratio in two groups. Ethical approval was sought from and granted by the local research ethics committee in October 2021 (Prot. No. 865/2021/Oss/IOR), registered with the acronym “CAL-KvsP”. Informed consent was obtained from all patients before the surgery was scheduled, following the principles of the Declaration of Helsinki. All study data were treated with maximum confidentiality.

### 2.1. Study Population

A retrospective data review on the IRCCS Istituto Ortopedico Rizzoli and Ospedale Maggiore “Pizzardi” database was performed to identify patients affected by intra-articular CF and surgically treated.

Exclusion criteria were age < 18 years at surgery, follow-up period < 2 years, deceased patients, different techniques from those under study, multiple fractures, foot and ankle surgery prior to the calcaneus fracture, cognitive retardation, alcohol or drug addiction, and chronic inflammatory joint diseases.

In total, 52 patients were eligible for the study. All patients were treated with open reduction and internal fixation for intra-articular CF by senior foot and ankle surgeons with extensive experience.

Two groups of consecutive patients were then formed, a Kirschner group (KG) that involved the use of fixation with Kirschner wires and a minimally invasive approach, and a plate group (PG) subjected to the use of the traditional dedicated plate and screws through an extensive approach.

### 2.2. Clinical and Radiological Assessment

Patients were clinically evaluated post-operatively at final follow-up, by using the Italian-validated American Orthopedic Foot and Ankle Society (AOFAS) Ankle-Hindfoot score for the clinical–functional assessment, the Italian-validated Short-Form 12 (SF-12) for the physical and psychological domain assessment, and the Visual Analog Scale (VAS) for pain [9,10,11,12].

A pre-operative clinical and functional evaluation was not performed, as per routine in the case of patients with acute fractures.

Using the Picture Archive and Communication System (PACS) software and Carestream Health (Rochester, NY, USA) software, radiographic evaluation was performed on pre-operative (conventional radiographs and CT scan) and post-operative outcomes at final follow-up (conventional radiographs), in order to primarily classify the fractures and create sub-groups, according to Sanders’ classification [13].

Complication and re-intervention rates have been registered.

### 2.3. Outcome Measures

We provided each patient recruited with a series of questionnaires assessing their state of health, including the American Orthopedic Foot and Ankle Society (AOFAS) Ankle-Hindfoot scale, the 12-item Short-Form Health Survey (SF-12), and the Visual Analog Scale for pain (VAS).

Each patient was classified on the basis of pre-operative CT scans in the coronal plane, according to Sanders’ classification [13]. Each group (KG and PG) was divided into sub-groups based on the classification of the fractures. This made it possible to assess the severity of the fractures and the complexity of their surgical reduction. Furthermore, each patient was asked whether he or she had any complications following surgery for CF or had undergone secondary operations, always related to the same traumatic event.

### 2.4. Operative Techniques

**Figure 1:**
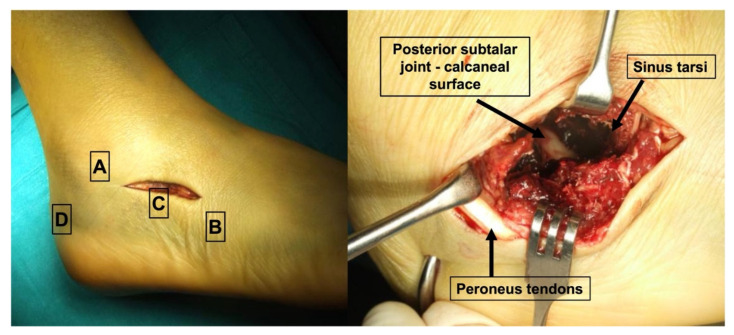
Mini-open posterior subtalar approach. On the left, (**A**) lateral malleolus apex, (**B**) cuboid bone, (**C**) sinus tarsi, (**D**) posterior calcaneal tuberosity.

K-wire fixation and mini-open approach. The technique involves a mini-open access to the sinus tarsi prosteriorly extended, which allows direct visualization of the posterior sub-talar joint. Using traction from the calcaneal tuberosity to reduce the fracture and to restore the calcaneal length and height, Kirschner wires are percutaneously positioned under fluoroscopic control [14] (Figure 1). At the end of the procedure, the limb is immobilized with the aid of a plaster boot. The plaster has to be maintained for approximately 4–6 weeks, without weight bearing. After 4–6 weeks, the percutaneous K-wires are removed as an outpatient procedure and a removable boot is positioned to begin active and passive mobilization and rehabilitation. Progressive weight bearing on the limb is allowed 8 weeks after surgery [4].

Dedicated plate fixation and extended approach. The surgical access to the lateral wall of the calcaneus (Ziup-Letournel “no-touch” approach) is characterized by an L-shaped incision, allowing easy access to the sub-talar joint and the calcaneal–cuboid joint. Reduction of the fracture fragments is then carried out. For the maintenance of the reduced bone fragments, Kirschner wires are used to maintain a provisional fixation before the definitive fixation with proper, dedicated plate and screws [15,16,17]. After surgery, a removable boot is positioned for 4 weeks. Progressive weight bearing is allowed 4 weeks post-operatively.

### 2.5. Statistical Analysis

All the variables used for describing the sample are continuous and they have been expressed in terms of mean and standard deviation (SD).

The analysis of variance for repeated measures was used to evaluate the trend of the clinical scores during the follow-up period. The single-way analysis of variance was used to evaluate the differences between inter-group values when the variables followed a normal distribution, applying the T-Student test. Otherwise, the non-parametric Wilcoxon rank test was used.

Normality of the distribution of the variables was examined applying the test of normality by Shapiro and Wilk.

For all the tests, a *p*-value < 0.05 was considered significant.

Statistical analysis was performed using the Statistical Package for the Social Sciences (SPSS) software, version 25.0 (IBM Corp. Released 2017. IBM SPSS Statistics for Windows, Version 25.0. Armonk, NY, USA: IBM Corp).

## 3. Results

A total of 52 eligible patients who underwent surgery from 2013 to 2020 were registered. Of these, 14 were excluded due to a lack of complete radiographic documentation. Thus, 38 patients (42 CF) met the inclusion criteria and were retrospectively evaluated and divided into two groups (KG and PG) consisting of 19 patients each. The patients examined consisted of 27 males (71%) and 11 females (29%), with an overall mean age of 50.2 ± 15.5 (38–81). Fifteen patients had a left CF (39.5%), 19 had a right CF (50%), and 4 patients had a bilateral CF (10.5%).

The overall mean follow-up was 59.4 ± 11.8 months. The analysis of the obtained data from the 38 patients examined provided an overall mean post-operative AOFAS score of 70.5 ± 11.4 (45–85), a mean post-operative SF-12 PCS score of 46.1 ± 8.8 (28.4–58), and an SF-12 MCS score of 52.9 ± 10.8 (24.3–68.6) at the final follow-up.

**Figure 2:**
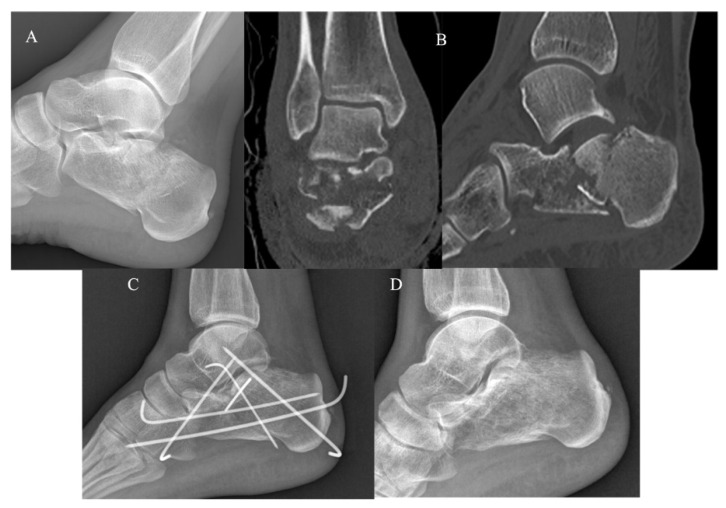
Mini-open approach and K-wire fixation. (**A**) Pre-operative radiographs; (**B**) pre-operative CT scan in coronal and sagittal plane showing a Sanders III CF; (**C**) post-operative radiograph control; (**D**) post-operative radiographs at 4-year follow-up.

**Figure 3:**
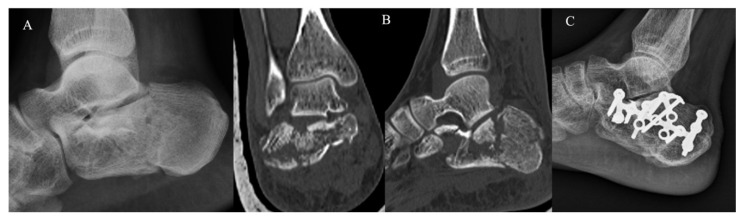
Extended approach and dedicated plate. (**A**) Pre-operative radiographs; (**B**) pre-operative CT scan in coronal and sagittal plane showing a Sanders III CF; (**C**) post-operative radiographs at 4-year follow-up.

The KG (Figure 2) and PG (Figure 3) consisted of 20 and 22 CF, respectively. The group demographic characteristics and specific mean final follow-up are summarized in Table 1.

At the final follow-up of 54.6 ± 16.8 months, the KG group (50% of the total pool) presented a mean post-operative AOFAS score of 70.7 ± 11.9 (45–83), mean SF-12 PCS score of 45.7 ± 6.8 (28.4–55.4), and SF-12 MCS score of 54.7 ± 9.9 (31.9–63.5). No patients underwent subsequent re-interventions and two patients (10.5%) presented minor complications (one case of delayed wound healing and one post-traumatic subtalar osteoarthritis).

At the final follow-up of 62.5 ± 16.3 months, the PG (50% of the entire pool) showed a mean post-operative AOFAS score at the end of follow-up of 70.1 ± 10.9 (46–85), a mean SF-12 PCS score at the end of follow-up of 46.5 ± 10.8 (28.9–58), and an SF-12 MCS score of 50.9 ± 11.8 (24.3–68.6). Nine patients (47.3%) underwent subsequent re-interventions, which, in all cases, involved removal of the hardware, due in six patients to intolerance (31.5%), and in three patients due to post-operative infection of the dedicated plate (15.7%). Finally, one patient underwent two reoperations, one year post-operatively to remove the hardware and two years later to perform a subtalar arthrodesis for severe post-traumatic osteoarthritis. The complications recorded in the PG (11, for a total of 57.8%) reflect almost exactly the number and type of re-interventions (six due to hardware intolerance, three post-operative local infections, two post-traumatic osteoarthritis).

No cases of delayed union or non-union were registered in either group.

The difference between the KG and PG post-operative clinical scores was found not to be statistically significant (*p* < 0.05).

The 42 fractures examined were classified according to the Sanders classification as 3 Sanders I fractures (7.1%), 17 Sanders II fractures (40.5%), 19 Sanders III fractures (45.2%), and 3 Sanders IV fractures (7.1%). The specific sub-groups’ outcomes and re-interventions are summarized in Table 2 and Table 3.

## 4. Discussion

CF have historically been a challenge for the orthopedic surgeon, particularly in the last century, with various attempts to optimize treatment and approaches [18,19]. Today, the best treatment and management of CF remains an object of debate [20,21,22,23].

The main findings highlighted in this retrospective study showed that the two surgical techniques investigated have achieved comparable clinical outcomes but a major discrepancy in the rate of re-intervention, at the expense of the use of dedicated plates and an extended lateral approach.

This evaluation had the purpose of comparing the clinical outcomes of patients affected by intra-articular CF treated with two different surgical approaches, at a minimum follow-up of 24 months (mini-open approach and percutaneous fixation with Kirschner wires vs. extended approach and internal fixation with dedicated plate and screws).

To obtain information about the superiority or non-superiority of one surgical technique over another, we adopted some of the most validated and used clinical scores, such as AOFAS, SF-12, and VAS. Since it was not possible to adopt hard end points, such as overall survival, due to the type of pathology, the main comparative parameter used was the patient’s quality of life following the trauma. The quality of life of the patients was analyzed by obtaining information on both subjective/objective and physical/mental parameters (AOFAS, SF-12 domains, VAS), as well as through the analysis of the sequelae of the trauma.

Of the 38 patients analyzed, 19 underwent surgery with a mini-open approach and percutaneous fixation with K-wires, and 19 underwent surgery with an extended approach and internal fixation with a dedicated plate. Through the analysis of the scores mentioned above, we were able to ascertain that more than 81% of the patients obtained a good post-operative AOFAS score (≥60 total points); up to 26% obtained an excellent score (≥80 total points) and obtained an overall average score of 70.5 ± 11.4. Analyzing the two groups (KG and PG) separately, we find that KG had good post-operative AOFAS scores in 80% of the cases and very good results in 30% of the cases, with an overall average score of 70.7 ± 11.9. Those who obtained the worst AOFAS scores (45–53) were patients who had factors that compromised at least part of the fracture-healing process (three patients). Of these, one had a bilateral fracture, one was of an advanced age (81 years), and one had risk factors (heavy tobacco smoker). They also had fractures classified as Sanders II and III. Giannini et al. reported, in their study published in 2016, a mean AOFAS score of 87.7 in patients treated percutaneously at a mean follow-up of 26.9 months, while Abdelgaid et al., in 2012, obtained AOFAS score results of 89.3 at 29 months of follow-up [4,24]. In contrast, Jin et al., in their study, achieved AOFAS scores of 84.4 at a mean follow-up of 16.9 months [25]. The discrepancy among our results and those of the cited studies can be partly explained by the higher mean age of our patients (54.4 ± 16.7) compared to 42.5 years in the Giannini study, 34.4 years in Abdelgait’s study, and 40.1 years in Jin’s study, and the much longer mean follow-up of our patients of 54.7 ± 16.8 months. Regarding the evaluation of psychometric scores, the patients in our study obtained an average post-operative score of 45.7 on the PCS-12, 54.7 on the MCF-12, and 3 on the VAS scale, confirming an overall good state of psychological and mental health, which can be deduced from the excellent MCF-12 score (Mental Component Summary). Finally, analyzing the re-intervention and complication rates of the KG, only 2 of 19 presented post-operative complications (10.5%) and none required re-intervention. On the other hand, Giannini et al. reported a complication rate of 13.4%, Jin et al. had 13.8% complications, and Abdelgait et al. had 0% complications; re-intervention rates were not reported [4,24,25].

In the PG, the patients achieved good post-operative AOFAS scores in 83% of the cases and very good in 22% of the cases, with a mean total score of 70.1 ± 10.9. Again, the two patients with the lowest AOFAS scores (46–50) had risk factors that impaired the normal fracture-healing process (smoking). Regarding the evaluation of psychometric scores, the PG patients in our study had average post-operative scores of 46.5 on the PCS-12, 50.9 on the MCF-12, and 3 on the VAS scale.

Wu et al., in a study published in 2012, reported a mean post-operative AOFAS score of 85.6 in patients treated with ORIF at a mean follow-up of 12 months, while Griffin et al., in their 2014 study, obtained a mean AOFAS score of 79.2, a mean PCS-36 score of 47.3, and an MCS-36 score of 53.4 at a mean follow-up of 18 months [6,26]. Analyzing the re-intervention and complication rates of the group of patients treated with ORIF, we obtained different results, as 2 of 19 patients (10.5%) presented post-operative complications (one patient presented wound dehiscence and one patient complained of severe post-traumatic subtalar osteoarthritis) and nine patients required re-interventions (47.3%). The removal of the hardware was due in six patients to intolerance (31.5%) and in three patients to infections of the metallic implant (15.7%). One patient underwent two re-operations. Wu et al. reported a complication rate of 33% in 148 patients, while Griffin et al. reported a complication rate of 20.3% and a re-intervention rate of 14.5% in 69 patients [6,26]. This discrepancy in results can be partly explained by the small number of patients in our study group, and by the average post-operative follow-up of the patients under study being 62.5 ± 16.3 months, which is much more indicative of the long-term quality of life of the patient compared to the period of 12 months in Wu et al.’s work and 18 months in the Griffin et al. study. It is also important to consider the inherent margin of error in the interpretation of clinical scores (AOFAS, SF-12, VAS) by patients, which may be more severe in a study with a small sample size.

The difference in the clinical scores between the two groups of patients (KG and PG) analyzed in this study was not statistically significant; as evidence of this, we find a *p*-value of 0.44 for the AOFAS scores, 0.66 for the PCS-12, 0.24 for the MCF-12, and 0.43 for the VAS score, greater than *p* > 0.05. On the other hand, the high rate of re-intervention that accompanies patients treated with the plate and screws (47.3%) is of great statistical importance. This leads to a significant lengthening of hospitalization times, a reduction in the perception of quality of life, and, lastly, a severe increase in care costs. These data must be taken into careful consideration with regard to the accurate choice of the type of surgical treatment, especially regarding the risk of re-intervention associated with similar final clinical results.

This study has some limitations, such as its retrospective nature and the small cohorts of patients enrolled. The absence of statistical significance, linked to the small sample examined, makes the quantitative results interesting but in need of future studies on larger cohorts. On the other hand, this study highlights the need to re-evaluate the use of temporary hardware such as Kirschner wires in the treatment of CF, despite not being considered the current gold standard. This is also in consideration of the easy availability (developing countries or emergency treatment) and the very low economic cost (linked both to the hardware itself and to the reduced need for re-intervention). Other recent studies confirm this minimally invasive trend in the treatment of intra-articular calcaneal fractures [27].

## 5. Conclusions

Although the percutaneous method is a less widely used technique than the ORIF one, an increasing number of studies are reported in the literature concerning minimally invasive techniques for the treatment of these fractures, because they present lower complication rates than those reported for standard open procedures.

The present study showed that, in the cases analyzed, the two surgical approaches used for the treatment of CF achieved comparable clinical outcomes, without statistically significant differences. The only substantial difference found between the two groups of patients was the re-intervention rate that afflicted them. In fact, compared with 0% of patients in the KG, we obtained a re-intervention rate of 47.3% in the patients treated with an extended approach and dedicated plate. 

In conclusion, the results of both treatments were satisfactory in the majority of patients examined. 

Prospective studies with larger patient cohorts and longer follow-up periods would be necessary to better evaluate the advantages and disadvantages of each technique. Eventually, given the non-univocal nature of the results, it would be useful to carry out a meta-analytical study summarizing the results of the hundreds of trials in the literature in order to better understand the superiority of one technique over the other.

## Figures and Tables

**Table 1:** Demographic characteristics and mean follow-up of KG and PG.

	K-Wire Group	Plate Group
**N° of patients**	19	19
**Sex (M/F)**	13/6	14/5
**Age (years)**	54.5 ± 16.7	45.5 ± 12.9
**Follow-up (months)**	54.7 ± 16.8	62.5 ± 16.3
**Side (L/R/bilateral)**	9/9/1	6/10/3

**Table 2:** KG and PG clinical outcomes and Sanders classification sub-groups.

	K-Wire Group	Plate Group	*p*-Value (*p* < 0.05)
**AOFAS**	70.7 ± 11.9	70.1 ± 10.9	0.48
**PCS-12**	45.7 ± 6.8	46.5 ± 10.8	0.64
**MCS-12**	54.7 ± 9.9	50.9 ± 11.8	0.24
**VAS**	3 ± 1.4	3 ± 2	0.43

**Table 3:** Sub-group classification, outcomes, and re-interventions based on Sanders grading.

	Mean Clinical Score	K-Wire Group	ORIF Group	*p*-Value
**Sanders I**	N° of patients	2	1	
	AOFAS	81 ± 0	77 ± 0	-
	PCS-12	45.7 ± 5.1	53.4 ± 0	-
	MCS-12	58.1 ± 7.7	56.8 ± 0	-
	VAS	3.5 ± 0.7	3 ± 0	-
	Re-interventions	0	0	
**Sanders II**	N° of patients	8	8	
	AOFAS	72.1 ± 11.7	67.9 ± 13.4	0.29
	PCS-12	48.4 ± 4.6	45.7 ± 11.7	0.32
	MCS-12	56.8 ± 7.8	51.1 ± 8.8	0.09
	VAS	2.1 ± 0.6	3 ± 2.3	0.75
	Re-interventions	0	4	
**Sanders III**	N° of patients	9	7	
	AOFAS	66.9 ± 13.2	74.4 ± 7.6	0.7
	PCS-12	43.1 ± 8.5	51.1 ± 8.3	0.75
	MCS-12	51.7 ± 12.3	49.4 ± 14.2	0.61
	VAS	3.7 ± 1.7	1.7 ± 1.4	0.17
	Re-interventions	0	4	
**Sanders IV**	N° of patients	1	2	
	AOFAS	72 ± 0	60.5 ± 3.5	-
	PCS-12	48.5 ± 0	32.7 ± 0.4	-
	MCS-12	59.1 ± 0	52.1 ± 23.4	-
	VAS	2 ± 0	6.5 ± 0.7	-
	Re-interventions	0	1	
